# Genomic and Phenotypic Agreement Defines the Use of Microwave Dielectric Spectroscopy for Recording Muscle Lipid Content in European Seabass (*Dicentrarchus labrax*)

**DOI:** 10.3389/fgene.2021.671491

**Published:** 2021-08-30

**Authors:** Gareth Frank Difford, Carlos Díaz-Gil, Albert Sánchez-Moya, Muhammad Luqman Aslam, Siri Storteig Horn, Bente Ruyter, Marine Herlin, Marilo Lopez, Anna Kristina Sonesson

**Affiliations:** ^1^Nofima, Ås, Norway; ^2^ABSA-Culmarex, Mallorca, Spain; ^3^Department of Cell Biology, Physiology, and Immunology, Faculty of Biology, University of Barcelona, Barcelona, Spain

**Keywords:** Distell fat meter, concordance, phenomics, agreement, lipid

## Abstract

Recording the fillet lipid percentage in European seabass is crucial to control lipid deposition as a means toward improving production efficiency and product quality. The reference method for recording lipid content is solvent lipid extraction and is the most accurate and precise method available. However, it is costly, requires sacrificing the fish and grinding the fillet sample which limits the scope of applications, for example grading of fillets, recording live fish or selective breeding of fish with own phenotypes are all limited. We tested a rapid, cost effective and non-destructive handheld microwave dielectric spectrometer (namely the Distell fat meter) against the reference method by recording both methods on 313 European seabass (*Dicentrarchus labrax*). The total method agreement between the dielectric spectrometer and the reference method was assessed by Lin’s concordance correlation coefficient (CCC), which was low to moderate CCC = 0.36–0.63. We detected a significant underestimation in accuracy of lipid percentage 22–26% by the dielectric spectrometer and increased imprecision resulting in the coefficient of variation (CV) doubling for dielectric spectrometer CV = 40.7–46% as compared to the reference method 27–31%. Substantial genetic variation for fillet lipid percentage was found for both the reference method (*h*^2^ = 0.59) and dielectric spectroscopy (*h*^2^ = 0.38–0.58), demonstrating that selective breeding is a promising method for controlling fillet lipid content. Importantly, the genetic correlation (*r*_g_) between the dielectric spectrometer and the reference method was positive and close to unity (*r*_g_ = 0.96), demonstrating the dielectric spectrometer captures practically all the genetic variation in the reference method. These findings form the basis of defining the scope of applications and experimental design for using dielectric spectroscopy for recording fillet lipid content in European seabass and validate its use for selective breeding.

## Introduction

The European seabass (*Dicentrarchus labrax*) is a major marine aquaculture species in the Mediterranean. As with many other aquaculture species, optimizing lipid deposition and depletion is a crucial element to controlling production efficiency and product quality (Janhunen et al., 2017; Besson et al., 2019). European seabass store the majority of their ingested energy as lipids in the perivisceral compartment (Dias et al., 2005) and to a lesser extent in liver and the muscle. Farmed European seabass typically have 4–10 fold higher muscle lipid content than wild European seabass, which may impair fillet quality (Alasalvar et al., 2002; Fuentes et al., 2010; Doan et al., 2017). Conversely, feed efficiency, an extremely valuable trait, is favorably correlated with increased muscle lipid content in European seabass, which is contrary to many other species like salmonids (Grima et al., 2010; Knap and Kause, 2018).

Dietary interventions for lipid content have shown mixed and often complex results. For instance, simply decreasing the dietary lipid content, decreases the liver lipid content but has limited effects on the muscle lipid content (Peres and Oliva-Teles, 1999). Whereas replacing fish meal with vegetable protein sources increases the muscle lipid content but has no effect on the liver lipid content (Dias et al., 2005; Torrecillas et al., 2017). Conversely, replacing fish oil with vegetable oil increases the liver lipid content but has limited effects on muscle lipid content (Dias et al., 2005; Torrecillas et al., 2017). Excess lipid in the perivisceral compartment constitutes a high cost slaughter waste, whilst excess lipid in the liver represents a health risk factor, with fatty liver disorder reported in European seabass (Kaushik et al., 2004) and Japanese seabass (Zhang et al., 2019). Not surprisingly, the control of lipid depletion and deposition in different tissues and organs is an active field of research in European seabass.

Substantial genetic variation has been reported for muscle lipid content in European seabass (*h*^2^ = 0.25–0.77), suggesting that selective breeding maybe an effective method to control muscle lipid content (Haffray et al., 2007; Saillant et al., 2009; Vandeputte et al., 2014; Besson et al., 2019). Moreover, genetic and phenotypic correlations to feed conversion ratio and body weight gain are favorable, suggesting improved muscle lipid content is economically beneficial (Grima et al., 2010; Doan et al., 2017; Besson et al., 2019). However, an on-going limitation for selective breeding is that the genetic parameters for this cost-efficient and non-lethal method to record muscle lipid content have not been genetically validated against the gold standard reference method.

The reference method for lipid content is chemical extraction of lipid using organic solvents such as ethyl acetate, first described by Folch et al. (1957). This measurement is deemed the “True value” as it is the most accurate and precise measurement from which all other methods are benchmarked (Bligh and Dyer, 1959). However, it is a destructive method as the fish and a sample of the muscle are sacrificed during the measurement, thus the fish measured cannot be used directly for breeding and the fillet will be downgraded. Other disadvantages are that it is time consuming, requires large volumes of solvent and is costly (55–74.5 Euro per sample), which can limit the numbers of fish recorded. The genetic evaluations of fillet lipid content in European seabass have relied on a non-invasive proxy method called the Distell fat meter (formerly the Torry fat meter; Distell Inc., West Lothian, Scotland).

The Distell fat meter does not record lipid content directly, instead it uses dielectric spectroscopy in the microwave region (2 GHz) with a strip line sensor (L × b × w, 80 × 25 × 10 mm) in contact with the tissue of interest to record subdermal moisture content (Kent, 1990). The dry matter content (inverse of moisture content) and lipid content are highly correlated and conserved across most fish species (Sørland et al., 2004), with correlations ranging from 0.79 in European seabass (Saillant et al., 2009) to 0.97 in North sea herring (*Clupea harengus*; Kent, 1990). The Distell fat meter makes use of a calibration dataset of fish or fillets recorded with both the fat meter and the reference method, usually around 30 or so sampled. The Distell fat meter is a highly portable, handheld device which can provide rapid measurements on site. Crucially this method is non-destructive to the fish, which means fish can be kept for breeding purposes after recording or repeatedly recorded during experiments (Norris and Cunningham, 2004; Janhunen et al., 2017). However, this method has shown some level of imprecision and inaccuracy that requires averaging of repeated measurements and a correction equation using the reference method (Saillant et al., 2009).

To the best of our knowledge the genetic parameters of fillet lipid content using the reference method have not been estimated in European seabass. Furthermore, the genetic correlation between dielectric spectroscopy predicted lipid and the true reference method lipid content have not been reported for any species, despite European seabass aquaculture companies reportedly including this trait in their breeding programs (Janssen et al., 2017). Knowledge of the magnitude and direction of the genetic correlation between “true” trait and the indirect proxy trait are crucial to ensuring genetic gain in the true breeding goal trait. Thus, the objectives of the current study are to (1) evaluate the phenotypic agreement between fillet lipid content predicted using dielectric spectroscopy and the reference method (2) evaluate their respective genetic relationships in European seabass.

## Materials and Methods

### Resource Population

A total of 749 European seabass from the 2016- and 2017-year classes of the ABSA-Culmarex aquaculture company (ABSA-Culmarex, Mallorca, Spain) were included in this experiment (Table 1). The 2016 year class was generated via a combination of natural spawning over three consecutive days in a broodstock tank containing 20 males and 17 females, as well artificial pair crossing between 3 females and 16 males. The 2017 year class was generated from five natural spawning events over four consecutive days in three broodstock tanks with a total of 90 females and 76 males. As per standard commercial practice, the batches of fertilized eggs were cultured in 360 L incubators. Larvae were stocked in 6 m^3^ larval tanks until a mean weight of approximately 10 g. These year classes were set out to sea in September 2017 and April 2018 in sea cages Murcia, Spain. Fish were fed *ad libitum* a commercial feed formulation with an approximate composition of 40–41% crude protein and 18% crude fat. Fish were harvested and slaughtered following the standardized harvesting methods of the aquaculture company over three consecutive days in November 2018 at IMIDA-Marine Aquaculture Station (IMIDA, Murcia, Spain) at a mean weight of 376 and 279 g for the 2016 and 2017 cohorts, respectively. Individual whole fish were recorded for weight (g), sex by visual inspection of the gonads and a fin tissue sample was taken and stored in absolute ethanol for genotyping, parentage analysis, and genetic analysis.

**TABLE 1:** Descriptive statistics and phenotypic (dis)agreement for lipid content traits recorded on European seabass.

**Descriptive statistics^1^**						
**Traits**	**Mean ± SD^2^**	**CV %**	**Min – Max**	**RMSEP (%)**	***R* ± SE**	**CCC ± SD**
***Cohort 2016* (*n* = 116)**						
Lipid_True_	9.0^a^ ± 2.8	27.8	3.5–15.9	1.9	0.61 ± 0.07	0.36 ± 0.05
Lipid_DSAve_	6.4^b^ ± 2.6	40.7	2.0–12.7			
***Cohort 2017* (*n* = 197)**						
Lipid_True_	8.9^a^ ± 3.0	33.7	2.3–20.9	2.1	0.71 ± 0.05	0.63 ± 0.04
Lipid_DSAve_	7.4^b^ ± 3.5	46.7	1.1–18.8			
***Cohort 2016 + 2017* (*n* = 313)**						
Lipid_True_	9.0^a^ ± 2.8	31.1	2.3–20.9	2.1	0.67 ± 0.04	0.53 ± 0.04
Lipid_DSAve_	7.1^b^ ± 3.2	45.5	1.1–18.8			

*^1^SD = standard deviation; CV = coefficient of variation; RMSEP = root mean square error of prediction; *R* = Pearson’s correlation; and CCC = Lin’s concordance correlation coefficient. ^2^ Different superscripts denoted statistical differences as (*p* < 0.05).*

### Genotyping and Parentage Assignment

Tissues samples were sent to Identigen for DNA extraction and genotyping (Identigen Ltd, Dublin, Ireland). The combined-species 60K SNP array for the European seabass and the gilthead seabream (the MedFish SNP chip), which was developed in a collaboration between the MEDAID^1^ and PERFORMFISH^2^ consortia was utilized (Penaloza et al., 2020). A total of 22,246 SNPs were available on European Seabass after setting a quality threshold at 93%. Following this, SNPs with a minor allele frequency smaller than 0.01 and deviations from Hardy-Weinberg Equilibrium with a *p* value <10^–7^ were filtered. Culminating in 18,050 SNP markers used for computing the genomic relationships.

The R package sequoia (Huisman, 2017) was used to identify sibling relationships between all 749 genotyped individuals and assigning them dummy parents since none of the broodstock were tissue sampled or genotyped. The Medfish SNP markers above were filtered with an additional requirement that minor allele frequency > 0.30 and a random sample of 400 SNPs were used. The number of iterations of sibship clustering was set to 30, maximum number of offspring for a single individual was set to 500, all other values were set to the default (Huisman, 2017). For the entire population of 749 genotyped individuals, it was possible to assign 732 individuals to at least one parent and 724 individuals to both parents. In total there were 97 families of which 59 were full sib families with both parents assigned, ranging in size from 117 individuals to 2 individuals with a median of 5 and the remainder were half sib families with a single individual or with only a single parent assigned. In total the 37 sires contributed to between 117 and 2 offspring with a median of 8 offspring per sire and 33 dams contributed to between 148 and 2 offspring with a median of 8 offspring per dam. There were 40 full sib families from the 2016 cohort and 19 families from the 2017 cohort. Of the 313 individual phenotyped for both methods (described below) 280 of the individuals could be assigned to a family and 54 of the 59 full sib families were represented.

### Lipid Measurement

In total, eight lipid traits were recorded and described chronologically below, seven of which are by dielectric spectrometry, but a distinction is made whether single or repeated measurements per fish are used directly or an average is taken per fish or per side of the fish. (1) The reference method lipid content (LipidTrue); (2) the average per fish based on the handheld dielectric spectrometer (LipidDSAve); (3) the handheld dielectric spectrometer with four repeated measurements per fish (LipidDSRep); (4) the handheld dielectric spectrometer with two repeated measurements per fish which are an average of the left and right side measurements (LipidDSRepLR); and (5–8) the handheld dielectric spectrometer with single measurements corresponding to the left anterior (LipidDSLA), left posterior (LipidDSLP), right anterior (LipidDSRA), and right posterior (LipidDSRP).

**FIGURE 1:**
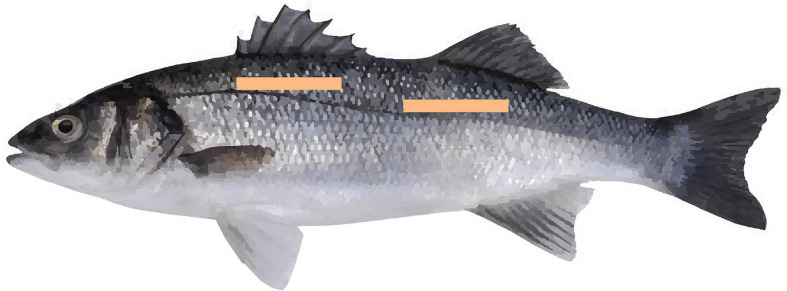
Diagram of the two positions the handheld dielectric spectrometer was used to record fillet lipid percentage. Note, four measurements were taken per fish with the two additional measurements mirrored on the other side of the fish (not shown).

Lipid was first estimated using the handheld dielectric spectrometer, namely Distell fat meter (Distell (Model-FM 692, www.distell.com). Four measurements were made on each of the 749 whole fish, over four body locations (left anterior, left posterior, right anterior, and right posterior) right above the lateral line (Figure 1). For all fish the same trained operator made the measurements and all fish had exactly four records. The estimated lipid percentage is predicted in real time by the instrument based on an internal calibration equation for European seabass, to which the operator has no control over. As the strip is 80 mm long, it was observed that on smaller fish the measurements on each side may partially overlap.

A random subset of 313 fish were filleted and both fillet samples were frozen at –20°C and sent for analysis at the Norwegian Institute of Food, Fisheries and Aquaculture Research (Nofima AS, Aas, Norway). The 313 fish came from 116 fish randomly sampled from the 2016 cohort and 197 from the 2017 cohort. Both fillets with skin on from each individual were homogenized and duplicate 100 g tissue samples drawn. Total lipids were extracted from each of 100 g duplicate samples using ethyl acetate as a solvent, according to the reference method (Folch et al., 1957). The average of the duplicates was the reference method lipid content value used for further analyses (LipidTrue).

### Statistical Analysis

#### Phenotypic Analysis

We assessed the agreement between the two methods on a phenotypic level and on a genetic level (described below). Assessing the phenotypic agreement between methods requires that both methods are used on the same individual, thus for phenotypic agreement Lipid_True_ and Lipid_DSAve_ comparisons were made on 313 individuals with both traits. To avoid potential population heterogeneity driving the correlation and thus the phenotypic agreement between the methods, we analyzed both within cohorts and combing cohorts for the phenotypic assessments (Difford et al., 2019).

The accuracy of the phenotypic prediction of Lipid_DSAve_ was tested against the reference method by means of paired *t*-tests. In addition, different agreement metrics were computed, including Pearson’s correlation coefficient, total variance, the coefficient of variation (CV), and the root mean square error. Concordance correlation analysis was conducted to evaluate the overall agreement between Lipid_DSAve_ and Lipid_True_, using Lin’s concordance correlation coefficient (CCC; Lin, 1989). The square root of the mean square difference between Lipid_True_ and Lipid_DSAve_ was used to calculate the root mean square error, a value which gives the average difference expected between a measurement from an alternative method and the true value. As an added validation step the difference between Lipid_True_ and Lipid_DSAve_ was plotted against body weight to check that residual difference were not related to body size (Supplementary Figure 1).

#### Genomic Analysis

On the contrary to phenotypic assessment, the genetic agreement between two methods allows including individuals with a single record with only one of the methods, we thus compared the genetic parameters on all 749 individuals having dielectric spectroscopy traits (Lipid_DSAve_, Lipid_DSLA_ Lipid_DSLP_, Lipid_DSRA_, and Lipid_DSRP_) with the subset of 313 individuals also having Lipid_True_. Furthermore, we included assessments on the repeated record level by having 2,996 from all four body positions recorded on 749 individuals (Lipid_DSRep_) and 1,498 from the average of the left and right-side measurements on 749 individuals (Lipid_DSRepLR_) to contrast repeatability estimates for this method.

The genetic parameters and variance components were estimated using univariate animal models with average information criterion restricted maximum likelihood in DMU version 6 (Madsen and Jensen, 2014). The model for Lipid_True_ and dielectric spectroscopy traits (Lipid_DSAve_, Lipid_DSLA_ Lipid_DSLP_, Lipid_DSRA_, and Lipid_DSRP_) had the following form:

(1) $$y_{\mathrm{ijk}}=\mu +C_{i}+S_{j}+a_{k}+e_{k}$$

Where *y_ijk_* is the lipid trait of the *k*th fish (*k* = 1–313) recorded in the *i*th cohort *C* (*I* = 1 or 2 for 2016 and 2017) which has the *j*th sex *S* (*j* = 1 or 2). Note, when analyzed within cohort the fixed term *C* is omitted. The random term *a* is the random additive genetics effects *a* ∼ ND (0, **G**σ^2^_*a*_), where **G** is the genomic relationship matrix derived from the Van Raden method one (VanRaden, 2008) and σ^2^_*a*_ the additive genetic variance. The random residual term e is the error of measurement *e* ∼ ND (0, **I**σ^2^_*e*_), **I** the identity matrix and σ^2^_*e*_ the residual variance.

The model for Lipid_DSRep_ and Lipid_DSRepLR_ had the following form:

(2) $$y_{\mathrm{ijklm}}=\mu +C_{j}+B_{l}+S_{m}+a_{k}+{\mathrm{pe}}_{k}+e_{i}$$

Where *y_ijklm_* is the *i*th recording (*i* = 4 for Lipid_DSRep_ and 2 Lipid_DSRepLR_) of muscle lipid percentage on the *k*th fish (*k* = 1–749) recorded in the *j*th cohort *C* (*j* = 1 or 2 for 2016 and 2017) and measured at the *l*th body site *B* (*l* = 1–4) with the *m*th sex *S* (*m* = 1 or 2). The random term *a* is the random additive genetics effects *a* ∼ ND (0, **G**σ^2^_*a*_), where **G** is the genomic relationship matrix derived from the Van Raden method one and σ^2^_*a*_ the additive genetic variance. The permanent environmental effect pe ∼ ND (0, **I**σ^2^_*pe*_), **I** the identity matrix corresponding to the *j* fish and σ^2^_*pe*_ the permanent environmental variance. The random residual term e is the error of measurement assumed to have a mean and distribution *e* ∼ ND (0, **I**σ^2^_*e*_), with **I** the identity matrix corresponding to the *l*th measurements and σ^2^_*e*_ the residual variance.

Heritability (*h*^2^) was calculated as the ratio of additive genetic variance to total phenotypic variance σ^2^_*a*_/(σ^2^_*a*_ + σ^2^_*e*_) for Eq. (1) and σ^2^_*a*_/(σ^2^_*a*_ + σ^2^_*pe*_ + σ^2^_*e*_) for Eq. (2). The repeatability (*t*^2^) is σ^2^_*a*_ + σ^2^_*pe*_/(σ^2^_*a*_ + σ^2^_*pe*_ + σ^2^_*e*_). The standard errors were derived by means of a Taylor series approximation.

A bivariate animal model was run between Lipid_True_ and all traits in Eq. (1) as well both repeated measures traits in Eq. (2) assuming the following variance structure across genetics, permanent environment, and residual variance structures:

(3) $$\text{Var}\left\lfloor \begin{array}{c} a_{1}\\ \begin{array}{c} a_{2}\\ \begin{array}{c} pe_{2}\\ \begin{array}{c} e_{1}\\ e_{2} \end{array} \end{array} \end{array} \end{array}\right\rfloor =\left[\begin{array}{ccccc} G\sigma_{a1}^{2} & G\sigma_{a21} & 0 & 0 & 0\\ G\sigma_{a12} & G\sigma_{a2}^{2} & 0 & 0 & 0\\ 0 & 0 & \text{I}\sigma_{pe2}^{2} & 0 & 0\\ 0 & 0 & 0 & \text{I}\sigma_{e1}^{2} & \text{I}\sigma_{e21}\\ 0 & 0 & 0 & \text{I}\sigma_{e12} & \text{I}\sigma_{e2}^{2} \end{array}\right]$$

Where subscripts 1 and 2 correspond to Eqs (1) and (2) above. Thus, no permanent environmental variance is defined for Eq. (1), as this method has no repeated records per individual. This is consistent with horizontal modeling, where parameters are estimated between a trait with a temporal or spatial trajectory and a static trait in livestock species (Shirali et al., 2017). Note when comparing Lipid_True_ and Lipid_DSA_ traits in Eq. (1) the random permanent environmental effect is omitted as there is only one phenotype per method per individual. The genetic correlations (*r*_g_) were estimated as the genetic covariance divided by the square root of the product of two variances, i.e.,

$r_{g}=\left(\sigma_{a12}\right)/\sqrt{\left(\sigma_{a1}^{2}\times \sigma_{a2}^{2}\right)}$.

The phenotypic correlation was estimated as

$r_{p}=\left(\sigma_{a12}+\sigma_{e12}\right)/\sqrt{\left(\left(\sigma_{a1}^{2}+\sigma_{e1}^{2}\right)\times \left(\sigma_{a2}^{2}+\sigma_{pe2}^{2}+\sigma_{e2}^{2}\right)\right)}$.

## Results

### Phenotypic (Dis)agreement Between Methods

**FIGURE 2:**
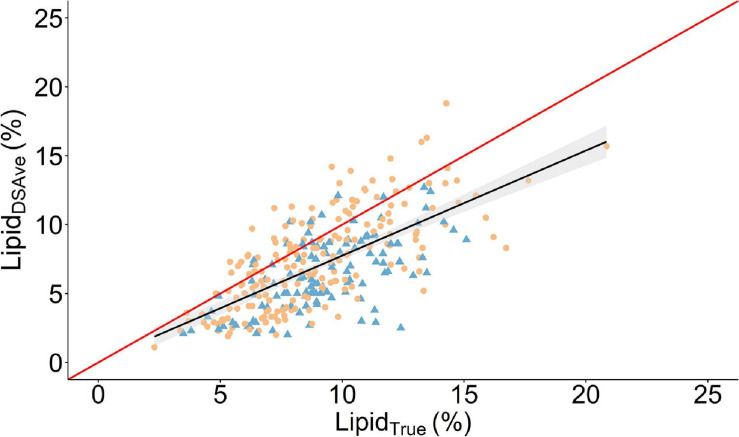
Scatter plot of the relationship between the mean fillet lipid percentage of four measurements using a handheld dielectric spectrometer (Lipid_DSAve_) and the laboratory reference method (Lipid_True_). A linear regression is given as the solid black line with the 95% confidence interval is gray shading. The solid red line is the line of unity. Round orange points denote 2017 cohort and triangle blue points are the 2016 cohort.

The descriptive statistics for the two methods can be found in Table 1 both within and across cohorts and the combined cohorts are visually presented in Figure 2. In general, Lipid_DSAve_ was underestimating the mean lipid content within and across cohort by 21–28.8% (*P* < 0.001). The standard deviation was larger for Lipid_DSAve_ than Lipid_True_ and the CV was substantially larger for Lipid_DSAve_ (Table 1). The correlations between methods were consistently moderate to high and positive but were significantly different from unity (*P* < 0.001). As the calibration equation for lipid content in European seabass was developed in an independent calibration set, we make the distinction that the root mean square error estimated herein is actually a root mean square error of prediction (RMSEP) and therefore, a good estimator on the error on measurement to be expected in independent populations. The RMSEP ranged from 1.9 to 2.1% within and across cohorts. The overall agreement was low to moderate as seen by the CCC ranging from 0.36 in the 2016 cohort to 0.63 in the 2017 cohort.

### Genetic (Dis)agreement Between Methods

**FIGURE 3:**
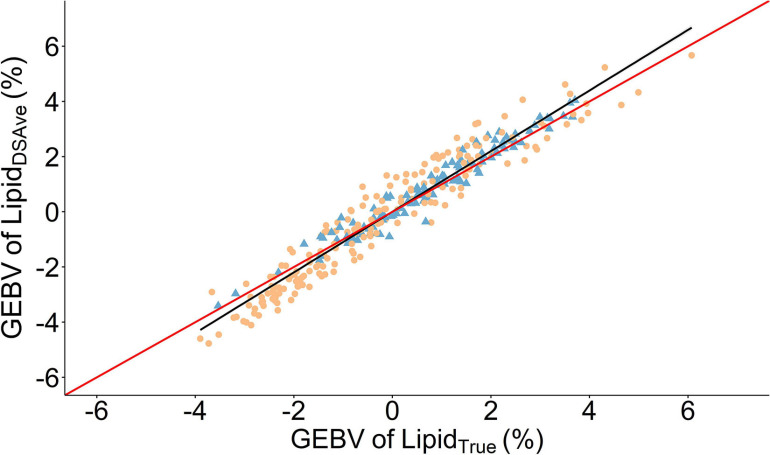
Scatter plot of the relationship between genomic estimated breeding values (GEBVS) of the mean fillet lipid percentage of four measurements using a handheld dielectric spectrometer (Lipid_DSAve_) and the laboratory reference method (Lipid_True_). A linear regression is given as the solid black line with the 95% confidence interval is gray shading. The solid red line is the line of unity. Round orange points denote 2017 cohort and triangle blue points are the 2016 cohort.

Lipid_True_ was significantly and substantially heritable 0.59 in the combined cohort (Table 2) as well as across cohort (Supplementary Table 1). The heritability estimates were significantly different from zero for (*h*^2^ = 0.38–0.40) across all four body sites and all had strong positive genetic correlations with Lipid_True_ *r*_g_ = 0.81–0.91 and can be visually appraised in Figure 3. The heritability increased with averaging of the four measurements from *h*^2^ = 0.40 for Lipid_DSRep_ with no averaging to *h*^2^ = 0.53 for Lipid_DSRepLR_ when averaging left and right sides to *h*^2^ = 0.59 for Lipid_DSAve_ when all four measurements are averaged. Similarly, repeatability increased from *t*^2^ = 0.56 with Lipid_DSRep_ to *t*^2^ = 0.80 for Lipid_DSRepLR_. Interestingly the genetic correlation to Lipid_True_ was very consistent irrespective of averaging *r*_g_ = 0.96–0.97. The phenotypic correlations to Lipid_True_ (after linear absorption of fixed effects) were the lowest for single body site measurements *r*_p_ = 0.46–0.53, but similarly increased with averaging of measurements from 0.60 for Lipid_DSRep_ to 0.66 for Lipid_DSAve_.

**TABLE 2:** Genetic parameter between lipid content traits recorded on European seabass.

**Genetic parameters^1^**							
	***N* (*n*)**	**σ_*a*_^2^**	**σ_*e*_^2^**	***h*^2^ ± SE**	***t*^2^ ± SE**	***R*_g_ Lipid_True_**	***R*_p_ Lipid_True_**
***Cohort 2016 + 2017***							
Lipid_True_	313	4.15	2.89	0.59 ± 0.11	–	–	–
Lipid_DSLA_	749	3.61	9.1	0.40 ± 0.07	–	0.91 ± 0.07	0.53 ± 0.05
Lipid_DSLP_	749	6.19	10.2	0.38 ± 0.07	–	0.88 ± 0.08	0.46 ± 0.05
Lipid_DSRA_	749	3.42	5.33	0.39 ± 0.07	–	0.81 ± 0.09	0.51 ± 0.05
Lipid_DSRP_	749	6.39	11.0	0.37 ± 0.07	–	0.89 ± 0.08	0.49 ± 0.05
Lipid_DSRep_	749 (2,996)	5.44	6.00	0.40 ± 0.14	0.56 ± 0.02	0.96 ± 0.03	0.60 ± 0.03
Lipid_DSRepLR_	749 (1,498)	5.32	1.87	0.53 ± 0.12	0.80 ± 0.01	0.95 ± 0.03	0.63 ± 0.04
Lipid_DSAve_	749	4.56	3.35	0.58 ± 0.12	–	0.96 ± 0.03	0.66 ± 0.03

*^1^*N* = number of fish; *n* = number of observations; σ_*a*_^2^ = additive genetic variance; σ_*e*_^2^ = residual variance; *h*^2^ ± SE = heritability estimate and standard error thereof; *t*^2^ ± SE = repeatability estimate and standard error thereof; *R*_*g*_ Lipid_*True*_ = genetic correlation with Lipid_*True*_; and *R*_*p*_ Lipid_*True*_ = phenotypic correlation with Lipid_*True*_ after linear absorption of fixed effects.*

## Discussion

### Phenotypic (Dis)agreement of Fillet Lipid Content

A number of studies have compared the lipid content estimated using dielectric spectroscopy and the reference method in fish species including European Seabass (Haffray et al., 2007; Saillant et al., 2009), salmonids (Hendry and Beall, 2004; Hanson et al., 2010; Caldwell et al., 2013), various freshwater species (Van Sang et al., 2009; Mesa and Rose, 2015), and marine pelagic species (Vogt et al., 2002; Bransden et al., 2007). However, these studies report different statistical metrics, although typically relying on Pearson’s correlation (*R*) or its square, the coefficient of determination (*R*^2^) from a linear regression, which ranged from (*R* = 0.31 to 0.92) across studies and species. This broad range of correlations supports the range of *R* = 0.61–0.71 found in the present study. However, these metrics are measures of linear association and fail to account for differences in mean (accuracy) or variance (precision) (Bland and Altman, 1994). In other words, two methods may be perfectly correlated but have completely different means and variances, which can have drastic effects of the conclusions drawn from an agreement study. This has led to many researchers estimating combined agreement indices, such as Lin’s CCC, which takes the linear association between two methods and penalizes this for deviations in accuracy and precision (Lin, 1989; Barnhart et al., 2007b). In this way, agreement is assessed considering all three facets of measurement and provide an index between 0 showing complete disagreement and 1 showing perfect agreement.

In the present study, we compared Lipid_True_ to Lipid_DSAve_ in a substantial number of European seabass (*n* = 116–313) and found the phenotypic agreement between the two methods to be low to moderate with CCC ranging from 0.36 to 0.63 across cohorts. Previous comparisons between the dielectric spectrometer and the reference method have not calculated the CCC or reported the means and variances of both methods and their correlation needed for us to retrospectively calculate the CCC. However, we were able to calculate the CCC between magnetic resonance imaging and dielectric spectrometry in Rainbow trout (*Oncorhynchus mykiss*) from Blay et al. (2021), which was 0.59. Furthermore, the CCC values herein are lower or comparable to that obtained for fillet lipid content in Atlantic salmon (*Salmo salar*) using a commercial near infrared (NIR) scanner on whole fillet and the reference method (CCC = 0.63) or a laboratory based NIR system on ground samples (CCC = 0.68) with the reference method (Difford et al., 2021). However, they are substantially less than that reported for Raman spectroscopy predicted lipid content compared to the reference method in Atlantic salmon CCC = 0.92 (Difford et al., 2021). Whilst the agreement between dielectric spectroscopy and the reference method appears to be lower than the majority of aforementioned studies, it is the only method which does not require sacrificing the fish. In some applications where measuring fillet lipid percentage on live fish is needed, for example on valuable brood stock fish or for repeated recording fish over their production life, producers may be willing to overlook this level of disagreement provided the causes for disagreement between the methods is known.

When the CCC deviates from 1 and indicates some level of disagreement between methods, it is useful to look at the different sources of disagreement, as this can be used to refine the scope of applications and inform statistical design considerations. For example, imprecision or variance reduction can be done by taking an average of repeated measurements and accuracy can be remedied by updated calibration equations (Barnhart et al., 2007a). The accuracy of Lipid_DSAve_ was significantly different to Lipid_True_, which underestimated mean lipid content by 21–28.8% across cohorts. This inaccuracy is larger than previously reported in the studies which have used linear regression to estimate the relationships between dielectric spectroscopy and the reference method. For example, Saillant et al. (2009) reported a positive intercept of 0.493 in a population of 30 European Sea bass with an average Lipid_DSAve_ of 6%, indicating an underestimation of 8% (inaccuracy) compared to the reference method. Similarly, the technical manual on the manufacturer’s website reports two case studies in European Seabass both with 29 individuals each, where the positive intercept indicates an 8–13% differences in means between methods (Distell, 2011). These findings suggest that dielectric spectroscopy for fillet lipid percentage can be improved by using an in-sample calibration with the reference method to quantify and correct for the inaccuracy. The use of in-sample calibration equations with the reference method is widespread in vibrational spectroscopy, for example for lipids in the milk of dairy cows (Rutten et al., 2010) and lipid in the fillets of Atlantic salmon (Difford et al., 2021).

In addition, we observed that Lipid_DSAve_ was less precise than Lipid_True_ as seen by the larger standard deviations around the mean and consequently the CV are consistently far larger for Lipid_DSAve_ (CV = 40.7–46%) as compared with Lipid_True_ (27–31%). In the present study we took an average of four repeated measurements over four body positions of the fish in order to obtain a value representative of the total fillet lipid content. By analyzing these spatially repeated measurements we found Lipid_DSRep_ to be medium to highly repeatable (*t*^2^ = 0.56). When we further took the average of two measurements from the left and right side Lipid_DSRepLR_ the repeatability increased to (*t*^2^ = 0.80). This demonstrates that the precision can be increased by taking an average of the records taken per fish (i.e., reducing the residual variance). This can be achieved by either repeating measurements spatially (i.e., recording different body sites) or temporally (i.e., at multiple times points; Falconer and Mackay, 1996; Kause et al., 2006). Importantly, the proportional reduction in total variance due to averaging increased numbers of records is given by $100-100\times \left(\frac{1+t^{2}\left(n-1\right)}{n}\right)$, thus lower repeatability traits gain a proportionally higher reduction in residual variance and thus total phenotypic variance by increasing the number of records per individual.

This is best illustrated by the work of Janhunen et al. (2017) in European whitefish (*Coregonus lavaretus*) who took four dielectric measurements per fish over three seasons, the repeatability of spatially repeated samples ranged from 0.71 to 0.91 across the seasons (i.e., equivalent to Lipid_DSRep_). When the mean of these measurements was taken (i.e., Lipid_DSAve_) the repeatability across seasons increased to 0.91–0.98 (Janhunen et al., 2017). Considering one of the advantages of the handheld dielectric spectrometer used, is that extra measurements are rapid and taken at little to no extra cost, this is a highly effective method to increase the precision of measurements. In the case of smaller fish, the area recorded by the instrument on the surface of the fish may overlap between repeated measurements and this may reduce the value of repeated measurements. In the present study, the effect of body position was significant between measurement, however, we did not find a relationship between measurement error and size of the fish (Supplementary Figure 1). An added consideration when taking an average from multiple spatial records is that recordings are balanced such that a simple average gives equal weighting to each measurement taken on different body sites, in the event that recordings are unbalanced it is necessary to take weighted averages. Furthermore, in the case of sampling live fish there is a limit to how long the fish can be out of the water and remain viable, thus there is a practical trade-off between increasing number of replicate measurements per fish and the wellbeing of the fish which needs to be considered.

### Genetic Agreement of Fillet Lipid Content

One of the most promising applications of dielectric spectroscopy is in breeding and selection for fillet lipid content. A number of authors have reported significant heritability estimates for Lipid_DSAve_ in European seabass (*h*^2^ = 0.28–0.77; Haffray et al., 2007; Saillant et al., 2009; Doan et al., 2017; Besson et al., 2019) which fully encompasses the heritability estimates in the present study 0.37–0.66. Together these results provide strong evidence that Lipid_DSAve_ is a significantly heritable trait in European seabass. However, dielectric spectroscopy is not a direct measurement of lipid content, rather it is a direct measure of subdermal moisture content which is assumed to be strongly correlated to lipid content (Kent, 1990). For the first time, the present study estimated the heritability of true lipid content in European seabass fillets (*h*^2^ = 0.59 ± 0.11). In addition, the heritability of Lipid_DSAve_ (*h*^2^ = 0.58 ± 0.12) was very similar to Lipid_True_, and the additive genetic variances were similar in magnitude between the methods.

Lipid_DSAve_ is the trait most often used in practice, however, this is a simple average of four measurements taken over four body sites on the fish in order to approximate fillet lipid percentage. As an added validation step, we evaluated the genetic parameters of each of the four measurements independently (Lipid_DSLA_, Lipid_DSLP_, Lipid_DSRA_, and Lipid_DSRP_), all four measurements in a repeatability model (Lipid_DSRep_), the average of the left-side and right-side measurements in a repeatability model (Lipid_DSRepLR_) and the average of all four measurements (Lipid_DSAve_). This exercise confirmed that the each of the four independent measurements are heritable (*h*^2^ = 0.38–0.40). Furthermore, by increasing the averaging across traits the heritabilities increased from 0.40 Lipid_DSRep_ to 0.58 for Lipid_DSAve._ This further demonstrates that taking a mean of repeated measurements increases the precision (i.e., reduces the residual variance) and consequently increases the heritability estimates.

In addition, this provided an opportunity to assess the phenotypic and genetic correlations between methods. The phenotypic correlations in the present study ranged from 0.61 to 0.71 which are slightly lower that the 0.80–0.82 reported previously for European seabass and the reference method (Saillant et al., 2009; Distell, 2011); as well as lower than the phenotypic correlation of 0.83 between low-field NMR and the dielectric spectrometer in 200 European seabass (Haffray et al., 2007). These are in range of the correlation 0.71 found when comparing the dielectric spectrometer to mid infrared spectroscopy on 359 European whitefish (Janhunen et al., 2017), as well as the correlation of 0.71 found when comparing magnetic resonance imaging and the dielectric spectrometer in 1,379 Rainbow trout (Blay et al., 2021). The reason for lower phenotypic correlations in the present study are not known, but we can speculate that changes in the diets and genetic makeup of the fish since the manufacturer created the calibration equation may be the cause of the lower reproducibility we observed for this equation. Calibration equations usually perform poorer in independent validation sets, as is likely the case here where the calibration equation was developed by the manufacturer on a sample of 29 European seabass and the validation set is completely independent (Distell, 2011). It maybe possible for researchers and producers to improve the phenotypic correlation and overall agreement of the dielectric spectrometer, by using an in sample calibration equation generated through recording a sample of fish using both methods and the fat meters internal “custom calibration” setting (Distell, 2011).

Crucially, for genetic evaluations it is the genetic correlations between alternative and reference methods called the “break-even correlation,” traditionally set at 0.70–0.80 (Robertson, 1959; Mulder et al., 2006), which is used for genetic validation of a method. The genetic correlation between Lipid_DSAve_ and Lipid_True_ was very high (*r*_g_ = 0.96 ± 0.03). To the best of our knowledge this is the first non-salmonid species to have genetic correlations estimated between the reference method and an alternative method for muscle lipid content. The genetic correlations herein are in the range of other spectroscopic measurements compared to the reference method in Atlantic salmon, for example a non-invasive field NIR system (*r*_g_ = 0.91), a laboratory NIR system (*r*_g_ = 0.93) and a laboratory Raman system (*r*_g_ = 0.98; Difford et al., 2021). These findings demonstrate that almost all the genetic variation in Lipid_True_ is captured by the handheld dielectric spectrometer, validating its use in selection programs for fillet lipid percentage.

## Conclusion

Based on these findings dielectric spectroscopy has low to moderate phenotypic agreement with the reference method. For applications where needs for precision and accuracy are high such as nutritional or physiological studies, it is recommended to use the reference method. However, for applications such as grading fillets or live fish into contrasting groups, dielectric spectroscopy is feasible. Fillet lipid percentage is highly heritable in European seabass using both the reference method and dielectric spectroscopy. Furthermore, the dielectric spectroscopic prediction of fillet lipid content was highly genetically correlated to the reference method. This genetic correlation was higher when an average of several spatially repeated measurements were taken over the fish. For genetic evaluation purposes taking the average of spatially repeated measurements over the fish provides a practical phenotype almost genetically equivalent to the reference method.

## Data Availability Statement

Most of the data supporting the results are included within the article/Supplementary Material as additional files. However, due to competitive nature and the privacy policy of commercial breeding companies, it is not permitted to share phenotypic, sequence, and genotypic data. The data can be made available for specific queries (on agreement) through the corresponding author.

## Ethics Statement

Ethical review and approval was not required for the animal study because experimentation was conducted in strict compliance with the EU legal frameworks related to the protection of animals used for scientific research (Directive 2010/63/EU). No specific permit was needed since fish were reared using classical practices by industry and the capture and slaughter followed commercial procedures for food fish and all measurements were collected on fish post mortem.

## Author Contributions

GD analyzed the data and drafted the manuscript. MA prepared the genotyping files. SH assisted in analysis and data curation. AS, MH, and ML were involved in funding acquisition. AS, BR, MH, ML, CD-G, and AS-M were involved in the design of study, sampling, phenotype recording, and actively contributed in discussions. All authors read the manuscript, gave suggestions and comments for the improvement, and approved the final manuscript.

## Conflict of Interest

MH and ML are currently employed at ABSA Culmarex. The remaining authors declare that the research was conducted in the absence of any commercial or financial relationships that could be construed as a potential conflict of interest.

## Publisher’s Note

All claims expressed in this article are solely those of the authors and do not necessarily represent those of their affiliated organizations, or those of the publisher, the editors and the reviewers. Any product that may be evaluated in this article, or claim that may be made by its manufacturer, is not guaranteed or endorsed by the publisher.

## Footnotes

1
http://www.medaid-h2020.eu/

2
http://performfish.eu/

## Supplementary Material

Supplementary Figure 1 Difference between Lipid_True_ and Lipid_DSAve_ plotted against body weight. A linear regression is given as the solid black line with the 95% confidence interval is gray shading. Round points denote 2016 cohort and triangle point are the 2017 cohort.
