# Supplementary figures and images for: Genomic and Phenotypic Agreement Defines the Use of Microwave Dielectric Spectroscopy for Recording Muscle Lipid Content in European Seabass (Dicentrarchus labrax)
Source: Front Genet. 2021 Aug 30;12:671491. doi: 10.3389/fgene.2021.671491 (PMC8435770; doi:10.3389/fgene.2021.671491)

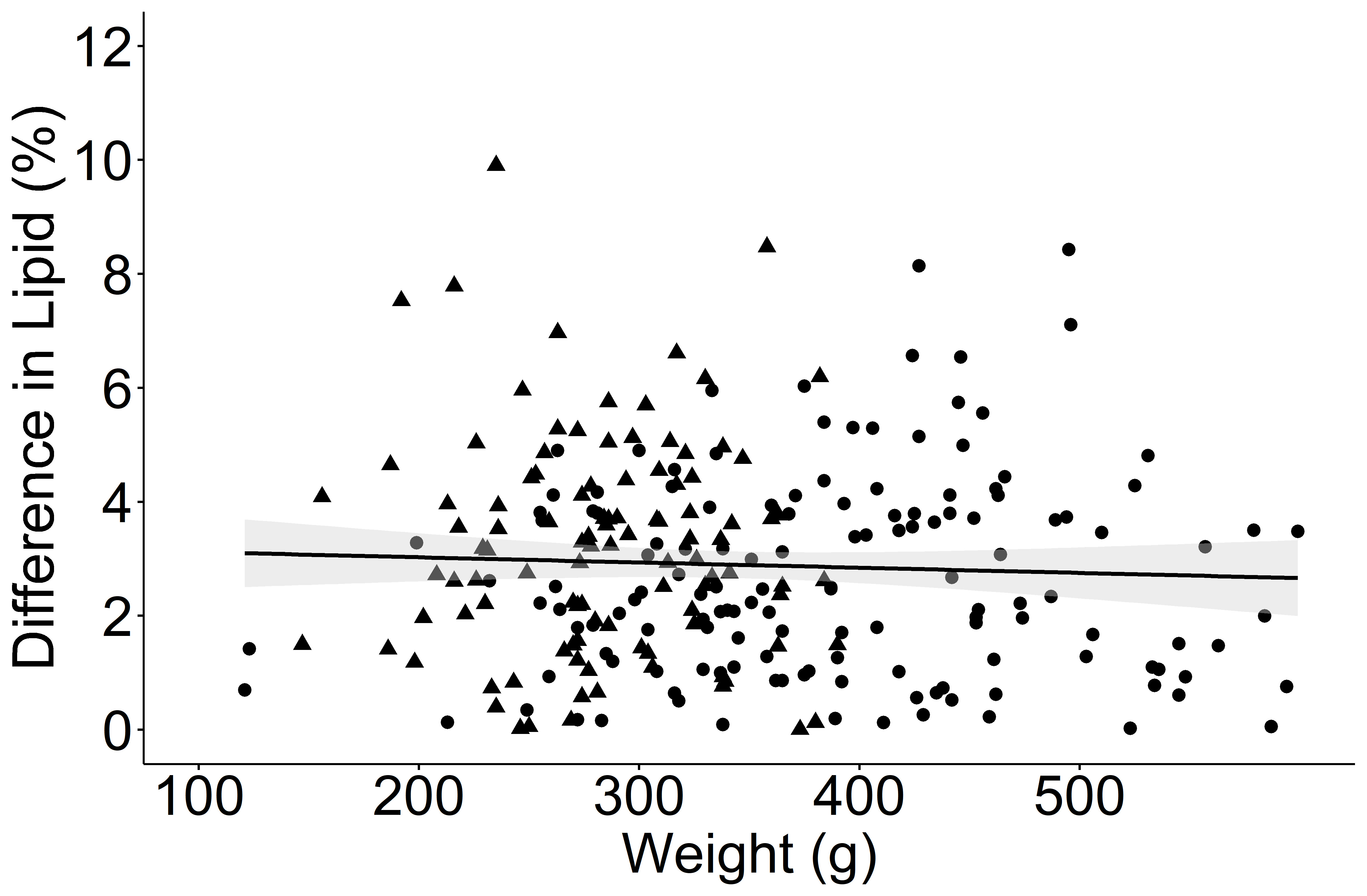

Supplement: Supplementary Figure 1 — Difference between LipidTrue and LipidDSAve plotted against body weight. A linear regression is given as the solid black line with the 95% confidence interval is gray shading. Round points denote 2016 cohort and triangle point are the 2017 cohort. [file Image_1.JPEG]
