# Supplementary material for: Genomic and Phenotypic Agreement Defines the Use of Microwave Dielectric Spectroscopy for Recording Muscle Lipid Content in European Seabass (Dicentrarchus labrax)
Source: Front Genet. 2021 Aug 30;12:671491. doi: 10.3389/fgene.2021.671491 (PMC8435770; doi:10.3389/fgene.2021.671491)
Supplement: Supplementary file 2 [file Table_1.DOCX]

**Supplementary Table 1**. Genetic parameter between lipid content traits recorded on European seabass.

| **Genetic parameters^1^** | | | | | | | |
| --- | --- | --- | --- | --- | --- | --- | --- |
|  | **N (n)** | **σ_a_^2^** | **σ_e_^2^** | **h^2^ ± SE** | **t^2^ ± SE** | **R_g_ Lipid_True_** | **Rp Lipid_True_** |
| *Cohort 2016* | | | | | | | |
| Lipid_True_ | 116 | 2.96 | 2.76 | 0.52 ± 0.23 | - | - | - |
| Lipid_DSAve_ | 200 | 2.08 | 3.50 | 0.37 ± 0.20 | - | * | 0.57 ± 0.07 |
| Lipid_DSRep_ | 200 (800) | 3.62 | 6.58 | 0.33 ± 0.25 | 0.41 ± 0.04 | 0.97 ± 0.10 | 0.52 ± 0.07 |
| *Cohort 2017* | | | | | | | |
| Lipid_True_ | 197 | 5.53 | 2.54 | 0.69 ± 0.13 | - | - | - |
| Lipid_DSAve_ | 549 | 6.30 | 3.23 | 0.66 ± 0.14 | - | 0.89 ± 0.07 | 0.69 ± 0.04 |
| Lipid_DSRep_ | 549 (2196) | 6.56 | 5.72 | 0.45 ± 0.15 | 0.61 ± 0.02 | 0.94 ± 0.04 | 0.63 ± 0.04 |

^1^ SD = standard deviation, CV = coefficient of variation, RMSEP = root mean square error of prediction, R = Pearson’s correlation and CCC = Lin’s concordance correlation coefficient. ^2^ Different superscripts denoted statistical differences as (p < 0.05).
